# Genomic and Functional Characterization of Longitudinal Pseudomonas aeruginosa Isolates from Young Patients with Cystic Fibrosis

**DOI:** 10.1128/spectrum.01556-23

**Published:** 2023-06-26

**Authors:** Courtney E. Chandler, Casey E. Hofstaedter, Tracy H. Hazen, David A. Rasko, Robert K. Ernst

**Affiliations:** a Department of Microbial Pathogenesis, University of Maryland—Baltimore, Baltimore, Maryland, USA; b Medical Scientist Training Program, University of Maryland School of Medicine, Baltimore, Maryland, USA; c Institute for Genome Sciences, University of Maryland School of Medicine, Baltimore, Maryland, USA; d Department of Microbiology and Immunology, University of Maryland—Baltimore, Baltimore, Maryland, USA; e Center for Pathogen Research, University of Maryland School of Medicine, Baltimore, Maryland, USA; University of Manitoba

**Keywords:** *Pseudomonas aeruginosa*, cystic fibrosis, airway adaptation, genomics, LPS evolution

## Abstract

Individuals with cystic fibrosis (CF) suffer from frequent and recurring microbial airway infections. The Gram-negative bacterium Pseudomonas aeruginosa is one of the most common organisms isolated from CF patient airways. P. aeruginosa establishes chronic infections that persist throughout a patient’s lifetime and is a major cause of morbidity and mortality. Throughout the course of infection, P. aeruginosa must evolve and adapt from an initial state of early, transient colonization to chronic colonization of the airways. Here, we examined isolates of P. aeruginosa from children under the age of 3 years old with CF to determine genetic adaptations the bacterium undergoes during this early stage of colonization and infection. These isolates were collected when early aggressive antimicrobial therapy was not the standard of care and therefore highlight strain evolution under limited antibiotic pressure. Examination of specific phenotypic adaptations, such as lipid A palmitoylation, antibiotic resistance, and loss of quorum sensing, did not reveal a clear genetic basis for such changes. Additionally, we demonstrate that the geography of patient origin, within the United States or among other countries, does not appear to significantly influence genetic adaptation. In summary, our results support the long-standing model that patients acquire individual isolates of P. aeruginosa that subsequently become hyperadapted to the patient-specific airway environment. This study provides a multipatient genomic analysis of isolates from young CF patients in the United States and contributes data regarding early colonization and adaptation to the growing body of research about P. aeruginosa evolution in the context of CF airway disease.

**IMPORTANCE** Chronic lung infection with Pseudomonas aeruginosa is of major concern for patients with cystic fibrosis (CF). During infection, P. aeruginosa undergoes genomic and functional adaptation to the hyperinflammatory CF airway, resulting in worsening lung function and pulmonary decline. All studies that describe these adaptations use P. aeruginosa obtained from older children or adults during late chronic lung infection; however, children with CF can be infected with P. aeruginosa as early as 3 months of age. Therefore, it is unclear when these genomic and functional adaptations occur over the course of CF lung infection, as access to P. aeruginosa isolates in children during early infection is limited. Here, we present a unique cohort of CF patients who were identified as being infected with P. aeruginosa at an early age prior to aggressive antibiotic therapy. Furthermore, we performed genomic and functional characterization of these isolates to address whether chronic CF P. aeruginosa phenotypes are present during early infection.

## INTRODUCTION

Cystic fibrosis (CF) is an autosomal recessive genetic disorder caused by mutations in the cystic fibrosis transmembrane conductance regulator (*cftr*) gene. Mutations in *cftr* lead to protein malfunction or loss of function, resulting in defective transport of chloride ions across epithelial surfaces (1, 2). This malfunction alters the nature of the airway surface liquid, leading to impaired mucociliary function, which directly impairs the noninflammatory host defense against inhaled microorganisms. As such, CF patients are more susceptible to microbial colonization and infection and suffer from repeated airway infections throughout their lifetime (1, 3).

The Gram-negative bacterium Pseudomonas aeruginosa is one of the most common causes of chronic infection, with almost one-third of CF patients being colonized with P. aeruginosa by the time they reach the age of 20 (4–6). Despite early and aggressive antibiotic intervention, P. aeruginosa infections persist and can eventually lead to respiratory failure, lung transplantation, and death (7).

**FIG 1:**
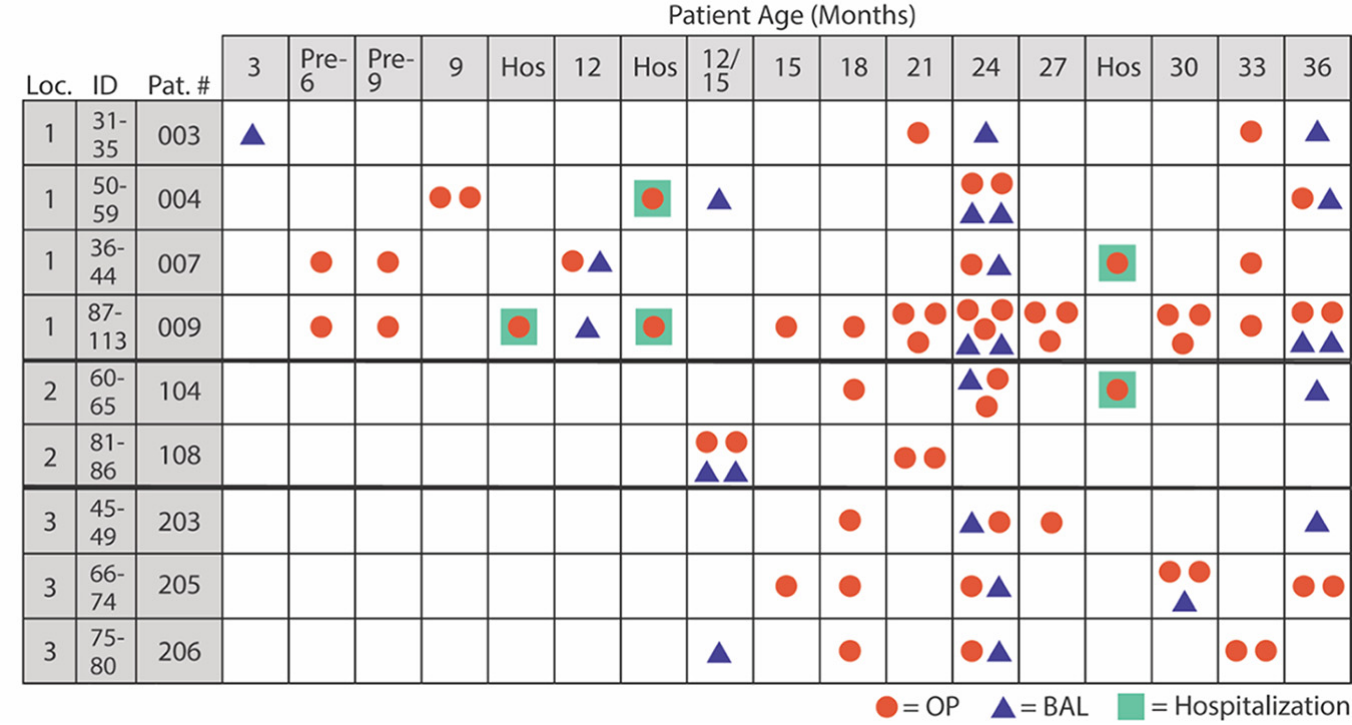
CF isolates from young children selected for sequencing. Isolates were selected from a total of nine different patients over time. In some cases, multiple isolates from the same time point were collected. OP, collection via oropharyngeal swab; BAL, collection via bronchial alveolar lavage; Hospitalization, samples collected during a hospital visit caused by an exacerbation event; Loc., location (region); Pat., patient. Region numbers: 1, Seattle, WA; 2, Houston, TX; 3, Cleveland, OH.

Within the CF airway microenvironments, P. aeruginosa genetically adapts to and evolves during chronic infection (4, 8, 9). Longitudinal studies have demonstrated that P. aeruginosa undergoes multiple genomic and phenotypic changes during chronic infection that are characteristic hallmarks of adaptation. These include palmitoylation of the outer membrane molecule lipid A, the loss of quorum sensing (QS), and an increased resistance to antibiotics (9). Although genomic sequencing studies have been conducted with P. aeruginosa CF isolates in the United States (10, 11), none have focused specifically on longitudinal isolates from patients within the first few years of life. Here, we sequenced 128 isolates of P. aeruginosa from the environment, CF patients, non-CF bronchiectasis patients, and patients with acute nonairway P. aeruginosa infection. Of these isolates, 81 are from nine CF patients in early childhood and were collected approximately every 6 months at routine well visits, in addition to any sick visits, over a 3-year period (Fig. 1) (12, 13). These isolates, from patients ranging in age from 3 months to 3 years, represent the earliest isolates from CF patients sequenced to date. Furthermore, these isolates would be difficult to acquire today due to early and aggressive antibiotic therapy, as well as the wide use of highly effective CFTR modulator therapies (e.g., the combination of elexacaftor, tezacaftor, and ivacaftor [ETI]), which improve CFTR function for those with specific *cftr* mutations and may impact the ability of P. aeruginosa to colonize the airway (12, 13).

In the current study, we examined CF airway adaptation phenotypes (e.g., lipid A modification, antibiotic susceptibility) alongside their associated genes. Importantly, P. aeruginosa lipid A structure in chronic CF airway infection is palmitoylated from constitutive activation of the lipid A biosynthetic enzyme PagP (14). As the genetic basis for this adaptive change has not yet been determined, we investigated single nucleotide polymorphism (SNP)-level changes in *pagP* and examined the genetic divergence in lipid A biosynthetic genes across the sequenced isolates. Furthermore, as these 81 CF isolates from young children were obtained from three distinct geographical regions, we investigated whether location of patient residence influences early genetic adaptation. Additionally, we included 474 previously sequenced CF P. aeruginosa genomes from a cohort in Denmark (8) to determine whether country of residence influences organismal adaptation. Overall, our studies provide insight into previously uninvestigated aspects of P. aeruginosa adaptation and provide a rich resource for researchers in the field.

## RESULTS

### P. aeruginosa CF isolates are distributed throughout the genomic landscape.

Direct comparison of all isolates revealed an average genome size of 6.545 million bases (Mb) (Table 1). CF and environmental isolate groups had similar genome sizes (6.675 and 6.609 Mb, respectively, compared to an all-isolate average). The GC content was consistent across all isolates (65.99% ± 0.45%). To examine total gene content and therefore, the degree of similarity between the genomes, a large-scale BLAST score ratio (LS-BSR) analysis was conducted (15). A total of 15,538 genes were predicted. The core genome (i.e., the set of genes present in every isolate) was quantified by using genes with an LS-BSR score of 1, representing 100% sequence identity (Table 1). Using the 128 sequenced isolates from this study (see Table S1 in the supplemental material) and two P. aeruginosa reference isolates (accession no. NC_002516.2 for P. aeruginosa PAO1 and NZ_CP017149.1 for P. aeruginosa isolate ATCC 15692), the core genome consisted of 3,645 genes (23.46%). This is a larger predicted core genome than those previously described for P. aeruginosa (between 1% and 15% of the total pangenome) (16, 17). Isolates from CF patients had an increased core genome size (31.84% for all CF infant and adult isolates). This increase in core genome size is unsurprising as each of the compared isolates is from the human airway, likely representing an adaptation to this infectious niche.

**TABLE 1:** Analysis of all newly sequenced isolates^*a*^

Isolate type (*n*)	Total no. of genes	Avg genome size (Mb)	Avg GC content (%)	Core genes		Unique genes	
				No.	%	No.	%
All (130)^*b*^	15,538	6.545	65.99	3,645	23.46		
Environmental (15)	11,097	6.675	65.99	5,109	46.04	1,113	10.03
PAO1 (11)^*b*^	6,091	6.218	66.44	5,881	96.55	7	0.11
Non-CF bronchiectasis (5)	7,991	6.393	66.38	5,272	65.97	224	2.80
CF							
All (89)	12,379	6.609	65.68	3,941	31.84	1,592	12.86
Child (81)	10,811	6.485	66.29	4,162	38.50	502	4.64
Adult (8)	10,135	6.732	65.06	4,968	49.02	1,091	10.76
Non-CF clinical acute (10)	10,166	6.704	66.15	5,185	51.00	660	6.49

a Core genes were identified as having an LS-BSR score of 1 (100% conservation). Unique genes were solely present in the indicated group of isolates.

b Two reference isolates were included (P. aeruginosa PAO1 accession no. NC_002516.2 and NZ_CP017149.1). The analysis was not changed when the reference isolates were excluded.

The isolates were separated into seven distinct groups based on source: environmental (15 isolates), PAO1 isolates (9 isolates), non-CF bronchiectasis (5 isolates), child CF (81 isolates), adult CF (8 isolates), all CF (89 isolates), and clinical acute (10 isolates). Two PAO1 reference sequences (accession no. NC_002516.2 and NZ_CP017149.1) were also used in the analysis, as genetic differences among PAO1 strains have been documented (18). Genes unique to only one group of isolates were identified (Table 1; Table S1). Unique genes were defined as genes that had an LS-BSR score of ≥0.8 (approximately ≥80% sequence identity) among the isolates of one group and a score of <0.1 (approximately <10% sequence identity) in all other isolates. The group of all CF isolates had the greatest percentage of group-specific unique genes (12.86%) compared to all other isolate groups. Isolates from adults with chronic CF infection (CF adult group [10.76%]) had a greater percentage of unique genes than isolates from young children (CF child group [4.64%]) (Table 1). Together, this supports the hypothesis that CF isolates acquire greater genetic variation over time as P. aeruginosa adapts to the lung and establishes chronic infection.

**FIG 2:**
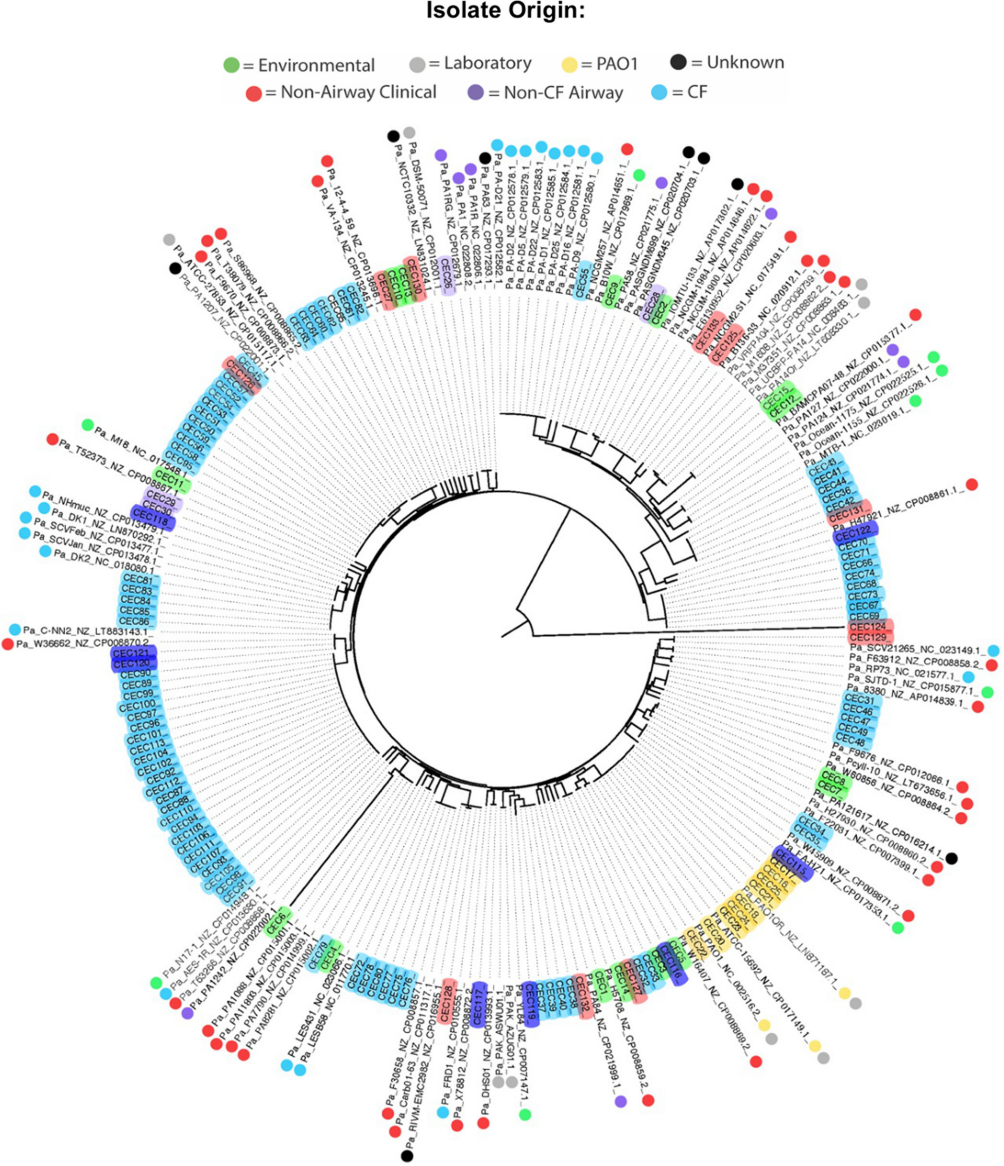
Phylogeny of newly sequenced P. aeruginosa isolates with available complete genomes of P. aeruginosa. All newly sequenced isolates are highlighted according to their sample origin (light blue, child CF isolate; dark blue, adult CF isolate; light purple, non-CF bronchiectasis; red, nonairway clinical disease isolates; yellow, PAO1; green, environmental). Downloaded whole-genome origins are indicated with circles along with their associated NCBI accession numbers.

We next conducted phylogenomic analysis to assess how these early CF isolates fit within the P. aeruginosa genomic landscape. Within the inferred phylogeny, several trends were observed (Fig. 2). All PAO1 sublines (yellow) clustered together into a single clade, highlighting their relative genetic similarity compared to the rest of the P. aeruginosa genomic landscape. The environmental isolates (green) were dispersed throughout the phylogeny, indicating they are not genomically distinct from disease-associated isolates. This supports the conclusions of previous studies that have demonstrated similarity between environmental and clinical isolates of P. aeruginosa, highlighting that each individual with CF likely acquires their P. aeruginosa isolate from their local environment (19). In many cases, isolates from a single CF patient (light blue) cluster together on a distinct clade. This observation further supports the understanding that P. aeruginosa acquired by an individual becomes hyperadapted to that specific individual’s airway and becomes increasingly dissimilar to other P. aeruginosa isolates, even during early infection and colonization, as shown in our CF young patient cohort. The adult CF isolates (dark blue) were more distributed throughout the phylogeny, likely because they originated from single isolates from multiple unique patients.

### Regional influence on genetic adaptation is limited.

**FIG 3:**
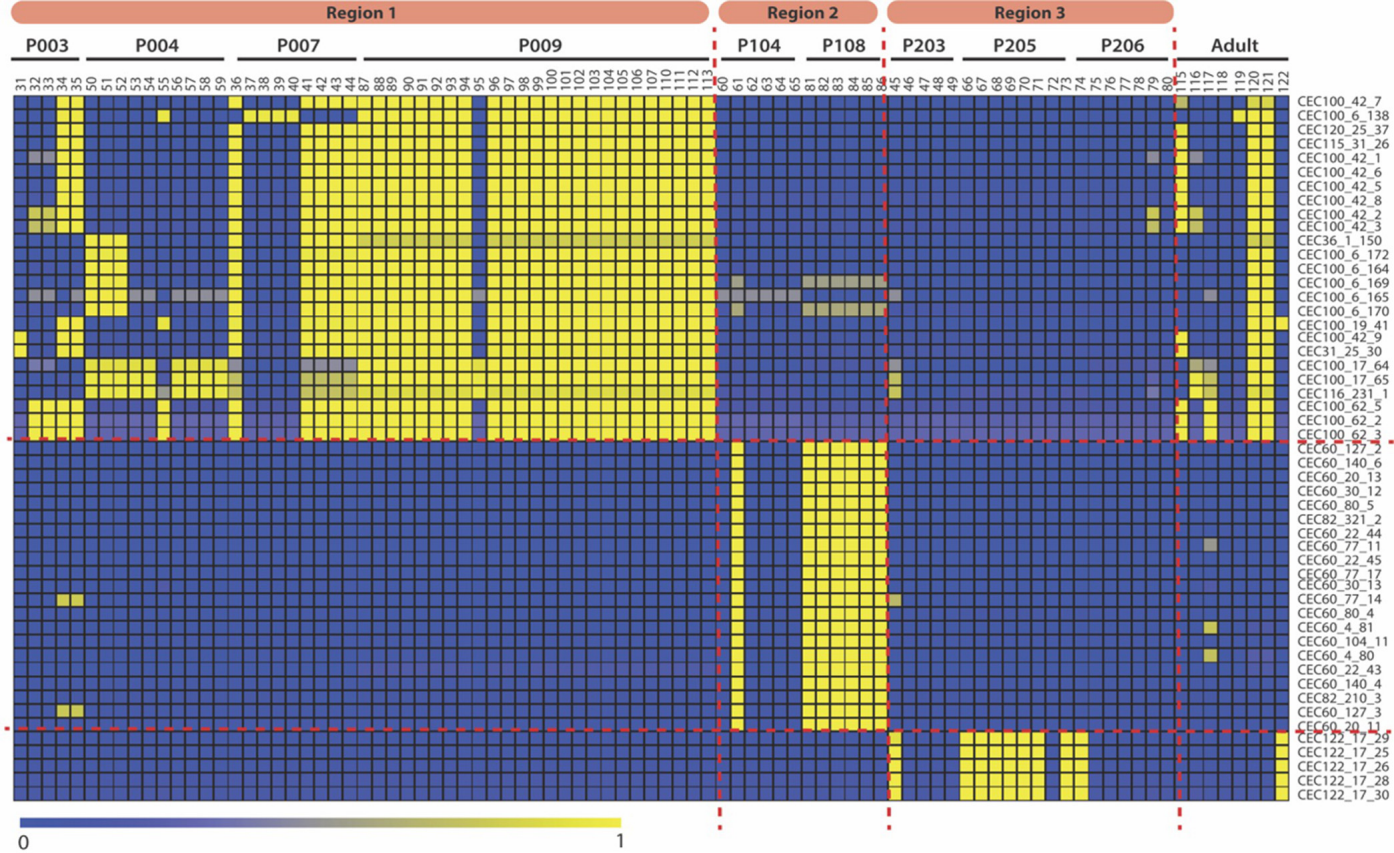
Visualization of location/region-specific genes. Raw LS-BSR values for region-specific genes were visualized in a heat map. Specific gene annotations are listed in Table S3 in the supplemental material. Many of the region-specific genes that were identified appear to also be patient associated.

Several previous studies have analyzed the geographical risk associated with P. aeruginosa acquisition, infection, and subsequent hospitalization in individuals with CF (20–22). However, none have analyzed if geography specifically impacts genetic adaptation in individual P. aeruginosa isolates. Here, we used our cohort of genomes P. aeruginosa isolates from young CF patients to investigate the impact of geography on P. aeruginosa genomic changes early in chronic infection. CF young child isolates (ID no. 31 to 113) were acquired from patients in three distinct geographical regions (collected in a previous study: region 1, Seattle, WA; region 2, Houston, TX; region 3, Columbus, OH) (12, 13). LS-BSR analysis was performed using CF young child and CF adult isolates (89 in total). In total, 13,410 genes were identified, and 9,663 genes were shared among the CF young child isolates, whereas 3,747 genes were specific to the CF adult isolates. These CF adult-specific genes were excluded because we did not have specific geographic locations for all adult samples. Region-specific genes were identified as any genes present (having an LS-BSR score of ≥0.8) in one region and not present (an LS-BSR score of <0.8) in any other region. Genes identified in each region were cross compared with the other regions to determine the amount of overlap and whether each region did or did not have unique genes that were not found in any other region (Table 2 and Fig. 3).

**TABLE 2:** Summary of regional overlap region-specific gene frequency^*a*^

Location (*n*)	No. of unique genes	No. (%) of genes shared with isolates from:			No. (%) of genes found by no. or % of isolates shown				
		Region 1	Region 2	Region 3	1 isolate	<10%	>50%	>80%	100%
Region 1 (49 [4 passages])	1,550		122 (0.9)	1,135 (8.5)	355 (22.9)	1,032 (66.5)	177 (11.4)	4 (0.025)	0 (0)
Region 2 (12 [2 passages])	309	122 (0.9)		140 (1)	8 (1.9)	9 (2.2)	297 (96.1)	0 (0)	0 (0)
Region 3 (20 [3 passages])	494	1,135 (8.5)	140 (1)		301 (60.9)	303 (61.3)	0 (0)	0 (0)	0 (0)

a Genes that were present in one region and absent in all other regions were identified from the LS-BSR data. Genes present in multiple regions (but not all regions) were also identified. Genes that were present in >50% of the number of isolates from a given region (or >45% for region 3) were removed from subsequent analysis. The majority of genes identified in regions 1 and 3 were in ≤10% of the isolates. Region numbers: 1, Seattle, WA; 2, Houston, TX; 3, Columbus, OH.

To further define how geographic region may influence adaptation, these genes were examined to determine the extent of their regional specificity. Table 2 summarizes the number of gene calls conserved among isolates in each region. Interestingly, not a single gene with 80% sequence identity was found in 100% of the isolates in any given region. Regions 2 and 3 did not have any genes that were present in 80% or more of the isolates from their respective regions either. Region 1 did have four genes that were conserved in 80% or more of the isolates, possibly due to the increased sample size for this location (Table 2).

The workflow to identify geographic-specific genes is represented in Fig. S1 in the supplemental material. This process resulted in 51 genes (25 specific to region 1, 21 specific to region 2, and 5 specific to region 3). Gene annotations were used to classify their predicted function and determine whether specific gene classes were implicated in regional adaptation. The gene calls, their functional annotations, and their functional classes are listed in Table S2. A heat map was used to visualize the LS-BSR values of region-specific genes (Fig. 3). Hypothetical genes were most frequently implicated in region-specific adaptation (9 in region 1 and 11 in region 2).

Finally, the majority of genomic studies on P. aeruginosa from CF patients have focused on isolates within one specific country. The largest of these studies sequenced 474 longitudinally collected isolates of P. aeruginosa from CF patients in Denmark (8). To determine whether country of residence significantly impacts P. aeruginosa adaptation, we conducted phylogenomic analysis of our sequenced CF isolates (all from the United States) and the sequenced isolates from Denmark to determine the degree of similarity between the isolates. We observed that isolates from the United States were generally distributed throughout the inferred phylogeny (Fig. S2 [U.S. isolates indicated with red dots]), suggesting that isolates originating from Denmark or the United States are not genetically dissimilar enough to be separated during phylogenetic analysis. Isolates originating from the same patient mostly clustered together (indicated in Fig. S2 by patient number), supporting our conclusion that the individual patient has a greater impact on genetic alteration than geography. Together, our data suggest geographic region and country of residence do not significantly induce specific genome-level alterations during P. aeruginosa adaptation.

### Pathoadaptive genes are variably represented throughout CF isolates.

Previous whole-genome sequencing studies of P. aeruginosa isolates from CF patients have identified many gene alterations thought to be selected for during chronic infection of and adaptation to the CF airway (23–25). In these studies, computational methods based on gene sequence dissimilarity were used to determine “pathoadaptive” genes in CF isolates compared to non-CF isolates. Notably, the gene lists in these independent studies were nonoverlapping, highlighting the individual and independent nature of adaptation (23–25). Using our unique cohort of young CF isolates, we queried for these previously identified pathoadaptive genes and used LS-BSR analysis to determine their presence or absence in each genome (Fig. S3). We selected specific genes to investigate based on phenotype data associated with the young child CF isolates obtained during the original study (12, 13) and their documented involvement in P. aeruginosa adaptation, including antibiotic resistance, quorum sensing, and lipid A modification. Our data indicate that many CF-related genes lack large-scale sequence divergence among the CF isolates from young patients. Adult CF isolates had slightly more genetic divergence across the queried genes (Fig. S3).

Several genes had multiple gene alleles, indicating that isolates had alternate forms of the same gene that arose via mutation but reside at the same locus in the genome. Most notably, *dnaX* had six alleles (Fig. S3 [*dnaX* alleles are marked with dots]). The *dnaX* gene encodes the gamma and tau subunits of DNA polymerase III (26). Allelic variants of *dnaX* may promote the mutator phenotype commonly observed in CF isolates (8, 10, 27), although we have not yet analyzed mutation rates with these isolates. Allelic variations were also observed in genes involved in motility (*pilB*, marked with “<”; *pilQ*, marked with “>”), long-chain fatty acid biosynthesis (*accC*, marked with “*”; *accE*, marked with “†”; *accF*, marked with “~”), and antibiotic resistance (*oprD*, marked with “$”; *mexR*, marked with “^”; *phzB1*, marked with “!”; *vrf*, marked with “–”) (Fig. S3) (28, 29).

### CF isolates display antibiotic resistance without changes to associated genes.

**FIG 4:**
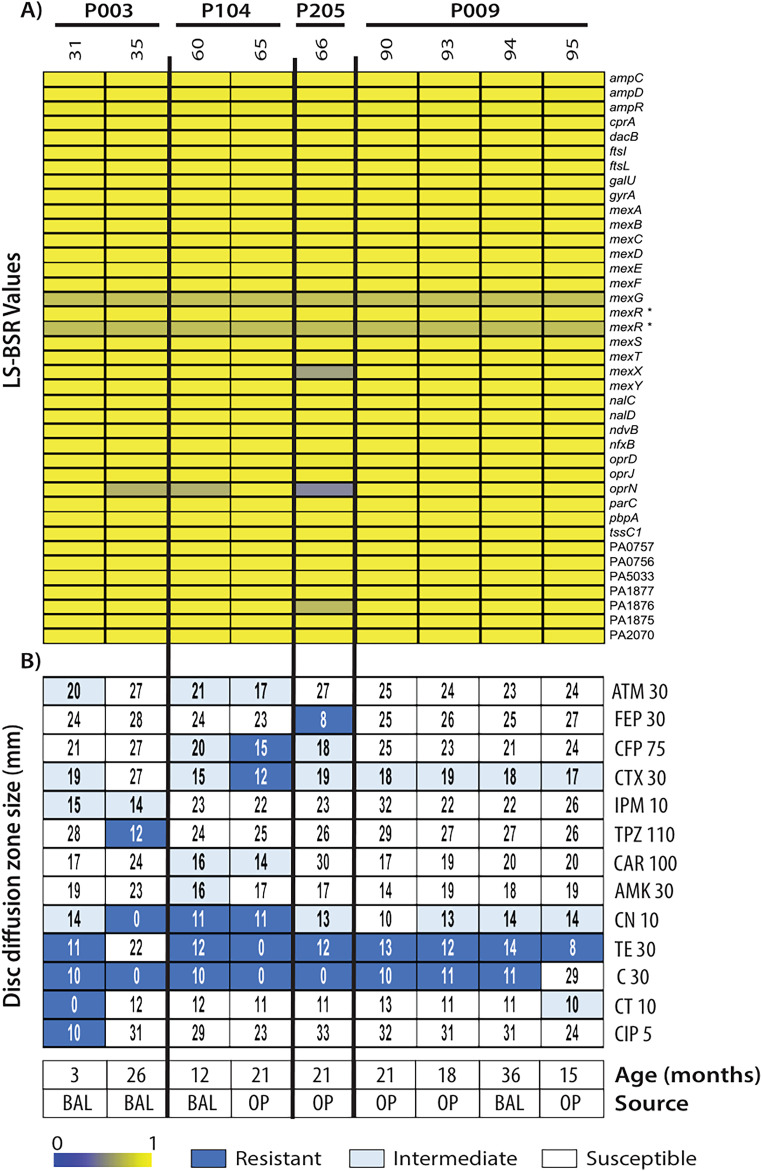
LS-BSR analysis of genes associated with antibiotic resistance and disc diffusion data. LS-BSR analysis of genes associated with antibiotic resistance (A) was coupled with disc diffusion data for several antibiotics (B): aztreonam (ATM 30), cefepime (FEP 30), cefoperazone (CFP 30), cefotaxime (CTX 30), imipenem (IPM 10), piperacillin-tazobactam (TPZ 110), carbenicillin (CAR 100), amikacin (AMK 30), gentamicin (CN 10), tetracycline (TE 30), chloramphenicol (C 30), colistin (CT 10), and ciprofloxacin (CIP 5). The numbers following the antibiotic abbreviations correspond to the antibiotic disc concentrations in micrograms. For reference, the patient age at the time of isolation and the isolation source are included. “*” indicates allelic variation.

Antibiotic susceptibility profiles were determined by disc diffusion in our cohort of young CF isolates (30). We investigated whether changes in antibiotic susceptibility could be explained by changes in antibiotic resistance-associated genes. Only a subset of isolates was previously analyzed by disc diffusion at the time of isolation, and therefore the genomic content of only these isolates was analyzed (Fig. 4). Our analysis revealed little genomic variability in most of the genes previously associated with antibiotic resistance (31), including the *mex* and *opr* operon genes involved in antibiotic extrusion (32). At the nucleotide level, isolate 66 (from patient 205) had genetic divergence in *mexX*, *oprN*, and antibiotic-associated ABC transporter system gene *PA1876* (defined by an LS-BSR score of <0.7) compared to all isolates in the cohort (33). The MexEF-OprN efflux pump has been associated with resistance to ciprofloxacin (34). However, this isolate was susceptible to ciprofloxacin (Fig. 4B [see CIP5]). Isolate 31, the only isolate with resistance to ciprofloxacin, had no detectable variation in the *mexEF* or *oprN* genes, suggesting *mexEF-oprN* gene regulation (or other gene involvement) dictates these antibiotic-resistant phenotypes. Isolates 35 and 60 also had genetic divergence in *oprN*, yet it was uncorrelated with changes to antibiotic susceptibility to any compound. However, there was no detectable genomic variation in *mexT*, a transcriptional regulator of the *mexEF-oprN* operon (Fig. 4A). In summary, our genomic analysis of antibiotic resistance-associated genes does not correlate with the observed phenotypic antibiotic susceptibility data.

### PQS production in CF isolates cannot be explained by genetic variation.

**FIG 5:**
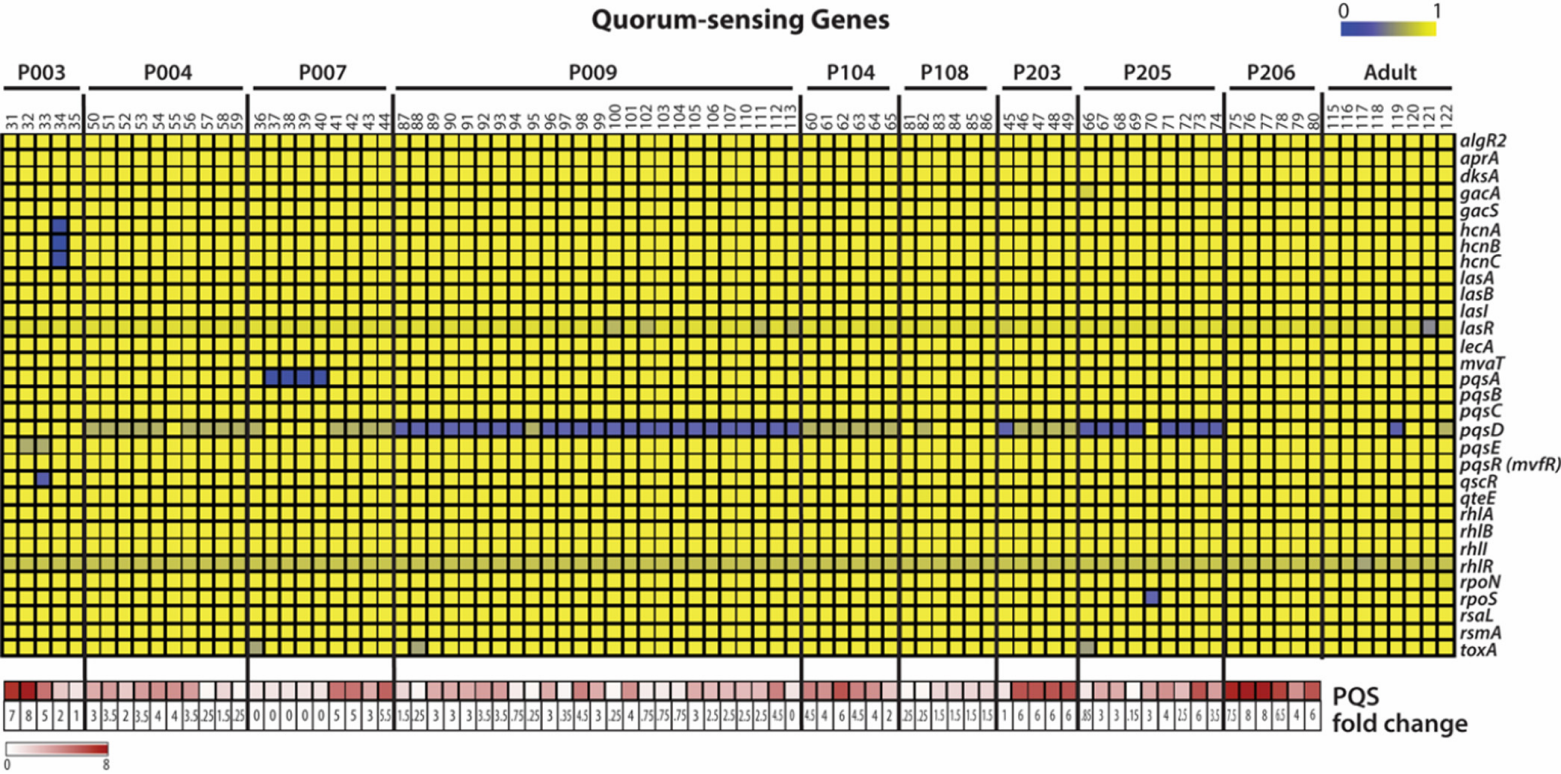
LS-BSR analysis of QS-related genes. The top array shows results from LS-BSR analysis of quorum sensing (QS)-related genes from CF isolates of P. aeruginosa (top array). QS genes were identified in a literature search. The bottom array indicates PQS production, as previously assayed from passage 2 isolates. Fold change is respective to a P. aeruginosa mutant that does not express PQS.

Loss of quorum sensing (QS) is a hallmark of P. aeruginosa adaptation within the CF airway (35–37). Pseudomonas quinolone signal (PQS) is one of the many QS molecules in P. aeruginosa involved in pathogenesis, and its synthesis is dependent on the *pqsABCDE* operon (38). In the CF airway, PQS downregulates host innate immune responses and is secreted in various amounts by P. aeruginosa isolates (39). Previously, PQS secretion was quantified for the CF isolates from young children by a liquid chromatography-mass spectrometry (LC-MS) method (40). The PQS secretion of the isolates was compared to that of a PQS-null mutant isolate to calculate a fold change over the reference null isolate. We examined QS genes using LS-BSR analysis and visualized the sequence identity scores as a heat map (Fig. 5). Genetic variation in PQS biosynthetic genes *pqsA*, *pqsD*, and *pqsE* was observed (Fig. S4). We additionally examined *lasR* and *rhlR*, transcriptional activators of virulence-associated genes, including QS genes, which are often mutated in CF isolates of P. aeruginosa (1, 8). Among the young child CF isolates, *lasR* had observable, but low genetic variability (LS-BSR score ratio of >0.6) in only four isolates from patient 009 (isolates 100, 102, 111, and 113), suggesting that *lasR* mutation is not a hallmark of early stage adaptation. The genetic sequences of *rhlR* and its associated regulator *rhlI* were consistent across all isolates. In summary, limited genetic variability was observed in QS-related genes in CF isolates from young children, which supports loss of QS as an adaptation associated with long-term chronic infection in older patients.

### Analysis of lipid A-related genes reveals limited genetic variation.

Lipid A is structurally altered during the transition from acute to chronic P. aeruginosa infection in CF patient airways (14, 41–43). Notably, a hexa-acylated lipid A structure results from activation of acyltransferase enzyme, PagP, a structure specifically associated with CF isolates (14, 42). Thus, the mechanism behind PagP overactivation is of interest as a cause has yet to be determined.

We examined all newly sequenced isolates to determine genetic variation in lipid A biosynthesis and modification genes using LS-BSR analysis (Fig. S5). We observed no nucleotide sequence divergence in the non-CF clinical isolates, and minimal divergence was observed in only one gene (*arnF*, required for aminoarabinose modification of lipid A) in environmental and non-CF bronchiectasis isolates. Interestingly, two divergent sequences were identified in *arnF*, indicating additional allelic variation (Fig. S5 [noted with *]). CF isolates showed little variation. Variations in *pagL* and *pagP* were seen in only two isolates (122 and 119, respectively).

To determine whether nucleotide-level variation is responsible for aberrant PagP activation, the canonical *pagP* sequence from Pseudomonas aeruginosa PAO1 (GenBank accession no. NC_002516.2) was used to examine our sequenced isolates using BLAST. The identified *pagP* sequences from all isolates were aligned, and sequence logos were generated to identify common SNPs (Fig. S6). The translated amino acid sequences of *pagP* associated with these SNPs revealed no divergence in the PagP active site residues (44, 45).

The lipid A structure of each isolate was investigated using matrix-assisted laser desorption ionization–time of flight mass spectrometry (MALDI-TOF MS) to determine whether the observed SNP-level differences impact PagP function. Lipid A mass spectra were obtained from the same culture that was used for sequencing (passage 4 for all CF isolates). The expected *m/z* in negative-ion mode for hexa-acylated lipid A resulting from PagP activity is *m/z* = 1,684 (*m/z* = 1,700 if the lipid A is doubly hydroxylated, *m/z* = 1,668 if there is no hydroxylation, or *m/z* = 1,604 for monophosphorylation) (Fig. S7 and Table S3); many CF isolates (64 total) did not have detectable PagP-specific hexa-acylated lipid A. These data suggest that the observed SNP-level variation was insufficient to induce lasting activation of PagP outside the context of the CF lung. We did not observe any genetic divergence in *phoP* or *phoQ* (regulators of *pagP* expression) (14). Alternatively, the *pagP* promoter region may be altered; however, the suspected loss of the hexa-acylated lipid A phenotype after multiple passages suggests that this likely has no significant influence on PagP activity.

## DISCUSSION

While several studies have described genetic adaptations of P. aeruginosa during infection of CF patient airways (4, 9–11, 46), the studies of patient populations in the United States lack longitudinal and cross-sectional isolates from infants and young children. Here, we provide the first multipatient, longitudinal sequencing study of P. aeruginosa isolates from young children. Whole-genome sequencing of 81 isolates from nine different patients, all aged 3 years or younger, provides a rich resource for the Pseudomonas and CF research communities. Eight additional isolates from adults with long-term P. aeruginosa infection were also analyzed, in addition to isolates from non-CF clinical presentations and various environmental contexts. The average genome size of the combined CF isolates was slightly higher than the average of other groups of isolates (6.609 Mb versus 6.545 Mb), including those isolated from the environment and laboratory-adapted PAO1 isolates. However, the genome size was comparable to that of non-CF clinical acute isolates, suggesting that disease-causing isolates of P. aeruginosa may undergo genetic variation to gain advantage within their infectious niche. P. aeruginosa has a highly plastic genome that is amenable to many genetic adaptations in these niches (47).

Analysis of all available whole-genome sequences of P. aeruginosa combined with our newly sequenced isolates revealed a distribution of our isolates throughout the P. aeruginosa genomic landscape. CF isolates from individual patient’s cluster together, but isolates from the environment and non-CF infections were dispersed among CF isolates. This observation further supports the notion that patients acquire P. aeruginosa from environmental reservoirs, and the divergence between environmental and CF-adapted isolates is not as significant as one might expect (19).

Of particular interest to membrane biologists is the structural alteration of lipid A during the transition from early to chronic infection. The emergence of hexa-acylated structure resulting from palmitoyl addition by the enzyme PagP is a well-documented adaptation in CF patient airways (14, 41, 44). Here, we investigated whether genetic changes in the P. aeruginosa isolates in our study could explain this lipid A phenotype. We demonstrate that while SNPs were present in various P. aeruginosa CF isolates, they did not correlate with phenotypic changes observed in lipid A structure when analyzed via mass spectrometry. Future studies using more sensitive methods such as gas chromatography to quantitatively analyze palmitoylated lipid A may be useful to complement the mass spectrometry data presented here. Lipid A was analyzed from the fourth passage of these isolates, suggesting that the hexa-acylated lipid A structure phenotype was lost or suppressed once cultured outside the selective pressures of the CF airway. It is likely that environmental conditions in the CF lung (e.g., oxygen concentration gradient, nutrient availability) trigger altered regulation of the PagP enzyme, potentially through the PhoP/Q two-component regulatory system (48, 49). Future transcriptional and proteomic studies of low-passage-number isolates from early infections may define the mechanism underlying PagP activation.

To date, the influence of geography at the genetic level has not been investigated within CF isolates of P. aeruginosa in the United States. While geography and climate are thought to influence acquisition of P. aeruginosa and patient prognosis once infected (22, 50), it was unknown whether geography would specifically influence colonization and adaptation. Here, we demonstrate that geography does not significantly influence gene-level or genome-scale alterations and that the selective pressures from the individual patient themselves appear to be a greater influence than geography. Within the United States, only 51 genes of 9,663 (~0.5%) were identified as being potentially region specific among three patient populations from the represented distinct regions. Of these genes, many were present in isolates from specific patients and were not distributed across all isolates from the region. This pattern suggests that geographic location has little impact on genetic adaptation. We also observed a similar trend when analyzing our 89 CF isolates versus 474 CF isolates from Danish patients (8). Isolates from different countries did not cluster separately, indicating their level of genetic similarity was not significantly influenced by country of origin; rather, clustering of isolates from the same patient was again observed.

In sum, our data suggest that region of residence does not meaningfully influence genetic adaptation of P. aeruginosa, both during early infection and at the stage of chronic infection. Taken together, our data provide the first genetic-level analysis of the hexa-acylated lipid A phenotype and influence of patient region of residence on the genome. Furthermore, even though CFTR modulator therapies (e.g., ETI) have revolutionized CF patient care, the microbiology of the CF airway does not change as drastically as anticipated (51–53). In this study, our analysis of archived young patient P. aeruginosa isolates offers a glimpse into early P. aeruginosa infection without *cftr*-directed or antimicrobial therapies. This work represents a significant data set for the Pseudomonas and CF research communities that will be foundational and serve as a baseline for future studies in the post-ETI era.

## MATERIALS AND METHODS

### Bacterial isolates.

Isolates were selected from a bank of P. aeruginosa isolates collected by Jane Burns, Margaret Rosenfeld, and Bonnie Ramsey (12, 13) and from the Ernst lab isolate bank (see Table S1 in the supplemental material). This study did not constitute human subjects research (as determined by the University of Maryland, Baltimore IRB [IRB no. HP-00099314]), as all patient data were deidentified prior to the strains being received in the Ernst laboratory. P. aeruginosa strains were isolated from various sources, including the following (Table S1): environmental isolates (*n* = 15), PAO1 laboratory sublines (*n* = 9, from Chandler et al. [18]), isolates from subjects with non-CF bronchiectasis (*n* = 5), CF young child (≤3 years old) isolates (*n* = 81), CF adult isolates (*n* = 10), and extrapulmonary, acute disease isolates (*n* = 10). Within the cohort of CF young children, isolates were obtained from subjects in three geographical regions: Seattle, WA (location 1), Houston, TX (location 2), and Cleveland, OH (location 3) (Table 2). All longitudinal CF isolates were obtained from subjects 3 years or younger; the earliest isolate was collected at 3 months of age. Isolates were collected by bronchial alveolar lavage (BAL) or oropharyngeal swab (OP). Patients are often infected with multiple isolates of P. aeruginosa (10, 54–56); therefore, multiple isolates (determined by colony morphology) from each subject were collected at one time point (Fig. 1).

The CF “adult” isolates were from subjects with chronic P. aeruginosa infection (average patient age, 15.49 ± 6.55 years) and were used to contrast the “early” isolates. Isolates from patients with non-CF bronchiectasis were collected to identify CF-specific influences of adaptation. All isolates were maintained in 20% glycerol stocks at −80°C prior to sequencing. For sequencing, all isolates were grown aerobically, and genomic DNA was isolated and used for whole-genome sequencing. During sequencing and analysis, three isolates (ID no. 19, 114, and 123) were removed as they were determined not to be P. aeruginosa based on quality checks of the data, contamination, or aberrant genome size of GC% and were therefore excluded from all subsequent analyses. Sample ID no. 108 and 109 were also excluded from analysis prior to sequencing due to missing culture stocks. Large-scale BLAST score ratio (LS-BSR) analysis was used to query the gene content in the total collection of genes (15).

For a complete list of isolates used in this study, see Table S1. All CF isolates were acquired during previous studies (12, 13) and have been maintained at −80°C. The cultures used for sequencing were passage 4.

### Preparation of genomic DNA.

Genomic DNA was isolated from aerobic culture grown at 37°C in lysogeny broth (LB) supplemented with 1 mM MgCl_2_ using a GenElute bacterial genomic DNA kit (Sigma-Aldrich, St. Louis, MO). The kit instructions were followed with the following exception: all DNA was eluted in 400 mL of ultrapure diethylpyrocarbonate-treated water (Thermo Fisher Scientific, Waltham, MA). The concentrations of all DNA preparations were determined using a NanoDrop 1000 spectrophotometer (Thermo Fisher Scientific, Waltham, MA). All preparations were stored at −20°C prior to sequencing.

### Whole-genome sequencing.

The genomes of all isolates analyzed in this study were sequenced as previously described (18). The sequencing libraries were generated with the Kapa HyperPrep kit (catalog no. KK8504) and sequenced on the Illumina HiSeq 4000 using a 2 × 150-bp paired-end kit. All software was used with default values. Raw sequencing reads were filtered to remove contaminating phiX reads using BBDuk, one of the BBTools software suite (sourceforge.net/projects/bbmap/). The raw reads were also filtered to remove contaminating Illumina adaptor sequences and quality trimmed using Trimmomatic v.0.36 (57). The resulting filtered reads were assembled using SPAdes v.3.13.0 (58). The resulting assemblies were then filtered to contain only contigs longer than 500 bp with a k-mer coverage of ≥5×. Genomes containing more than 500 contigs or an aberrant GC content were removed from further analysis.

### LS-BSR analysis.

The genomes of all 128 isolates or just the CF isolates (91 total) were compared using a large-scale BLAST score ratio (LS-BSR) as previously described (15, 59). The predicted protein-encoding genes of each genome that had ≥ 80% nucleotide sequence identity to each other were assigned to gene clusters using the UCLUST algorithm (60). Subsequently, representative sequences of each gene cluster were compared to the sequence of each genome using tblastn (61). Composition-based adjustment was turned off and the resultant tblastn scores were used to generate a BLAST score ratio (BSR) value indicating the detection of each gene cluster in each of the genomes. The BSR value was determined by dividing the score of a gene compared to a sequenced genome by the score of the gene compared to its own sequence. Heat maps of the LS-BSR values of the predicted genes were generated using the multiple-alignment viewing software MeV (62). The gene lists used in LS-BSR analysis and raw values generated for heat maps are listed in Tables S4 and S5.

### Phylogenomic analysis.

The genomes of our isolates were compared to previously sequenced complete Pseudomonas aeruginosa genomes available from GenBank before 1 January 2018 (all accession numbers are listed in Fig. 2) or to 474 draft whole-genome sequences of P. aeruginosa CF isolates from a prior study (8); the study authors, Søren Molin and Helle Johansen, kindly shared their assembled genome data with us. Comparison was conducted using an *in silico* genotyper (ISG) (63). Single nucleotide polymorphisms (SNPs) were detected relative to the completed genome sequence of reference P. aeruginosa isolate PAO1 (GenBank accession no. NC_002516.2) using the ISG, which uses the NUCmer (v.3.22) program (64) for SNP detection. SNP sites that were identified in all analyzed genomes were concatenated and used to construct a maximum likelihood phylogeny using RAxML (v.7.2.8) (65). The phylogeny was constructed using the general time-reversible (GTR) model of nucleotide substitution with the GAMMA model of rate heterogeneity and 100 bootstrap replicates. All phylogenies were visualized using the FigTree (v.1.4.2) program (http://tree.bio.ed.ac.uk/software/figree/).

### Disc diffusion and PQS analysis.

Disc diffusion and PQS analysis were conducted as a part of the original study during which the CF isolates were collected (12, 13).

### Multiple alignment and sequence logos.

*pqsA*, *pqsD*, and *pagP* sequences in individual genomes were identified by BLAST using the associated P. aeruginosa PAO1 sequence (downloaded from https://pseudomonas.com) in Geneious Pro software (https://www.geneious.com/). Sequences were aligned using ClustalOmega (https://www.ebi.ac.uk/Tools/msa/clustalo/). For *pagP* analysis, the Pearson/FASTA output was downloaded after ClustalOmega processing. AliView version 1.25 (http://www.ormbunkar.se/aliview/) was used to view the alignments and select the regions of interest. Sequence logos were generated using WebLogo (https://weblogo.berkeley.edu/logo.cgi) (66).

### Small-scale lipid A isolation and MALDI-TOF-MS analysis.

Lipid A was isolated from whole cells using an isobutyric acid-ammonium hydroxide-based extraction procedure as previously described (67). Cells from ~3 mL of culture left over after genomic DNA isolation (for sequencing) were centrifuged, and the supernatant was removed. Cell pellets were resuspended in 400 mL of 70% isobutyric acid and 1 M ammonium hydroxide at 5:3 (vol/vol). Samples were incubated at 100°C for 1 h, cooled on ice, and centrifuged for 5 min at 8,000 × *g*. Supernatants were transferred to a new tube and diluted 1:1 (vol/vol) with endotoxin-free water. Samples were flash-frozen on dry ice and lyophilized overnight. The dried material was washed twice with 1 mL of methanol, and lipid A was extracted in 100 mL of a mixture of chloroform-methanol-water (3:1:0.25 [vol/vol/vol]). One microliter of the extract was spotted onto a stainless steel MALDI target plate, followed by 1 mL of norharmane matrix (Sigma, St. Louis, MO) at a concentration of 10 mg/mL in 2:1 chloroform-methanol (vol/vol). All spots were allowed to air dry before MALDI-TOF MS analysis.

Lipid A was analyzed in negative-ion mode with reflectron mode on a Bruker microFlex (Bruker Daltonics, Billerica, MA) matrix-assisted laser desorption ionization–time-of-flight mass spectrometer. Data were acquired in negative-ion mode. The instrument was mass calibrated with an electrospray tuning mix (Agilent, Palo Alto, CA). Data were acquired with flexControl software and processed with flexAnalysis (v.3.4; Bruker Daltonics, Billerica, MA). All spectra were baseline smoothed before publication. The resultant spectra were used to estimate the lipid A structures present in each isolate based on their predicted structures and associated molecular weights.

### Gene overlap analysis.

To determine genes shared between sets of isolates, the online software Venny (v.2.1.0) was used (http://bioinfogp.cnb.csic.es/tools/venny/).

### Data availability.

All sequence data and genome assemblies generated in this study have been submitted to GenBank under BioProject no. PRJNA607994 (this study) and PRJNA490649 (18). The individual assembly accession numbers and Illumina sequence read accession numbers are listed in Table S1.
