# Supplementary material for: Genomic and Functional Characterization of Longitudinal Pseudomonas aeruginosa Isolates from Young Patients with Cystic Fibrosis
Source: Microbiol Spectr. 2023 Jun 26;11(4):e01556-23. doi: 10.1128/spectrum.01556-23 (PMC10433850; doi:10.1128/spectrum.01556-23)
Supplement: Supplemental file 1 — Supplemental material. Download spectrum.01556-23-s0001.pdf, PDF file, 3.0 MB [file spectrum.01556-23-s0001.pdf]

Supplemental Material

Genomic and functional characterization of longitudinal *Pseudomonas aeruginosa* isolates from young patients with cystic fibrosis

Courtney E. Chandler*[1], Casey E. Hofstaedter*[1,2], Tracy H. Hazen[3,4], David A. Rasko[1,3,4,5], Robert K. Ernst[1,4,5]

*These authors contributed equally to this work

1 – Department of Microbial Pathogenesis, University of Maryland – Baltimore, Baltimore, MD, 21201

2 – Medical Scientist Training Program, University of Maryland School of Medicine, Baltimore, MD, 20201

3 – Institute for Genome Sciences – University of Maryland School of Medicine, Baltimore, MD, 20201

4 – Department of Microbiology and Immunology, University of Maryland – Baltimore, Baltimore, MD, 21201

5 - Center for Pathogen Research, University of Maryland School of Medicine – Baltimore, MD 21201.

Supplemental Table 1. Isolates examined in this study. All *P. aeruginosa* isolates analyzed in this study are listed, including sample origin and DNA sequencing statistics.

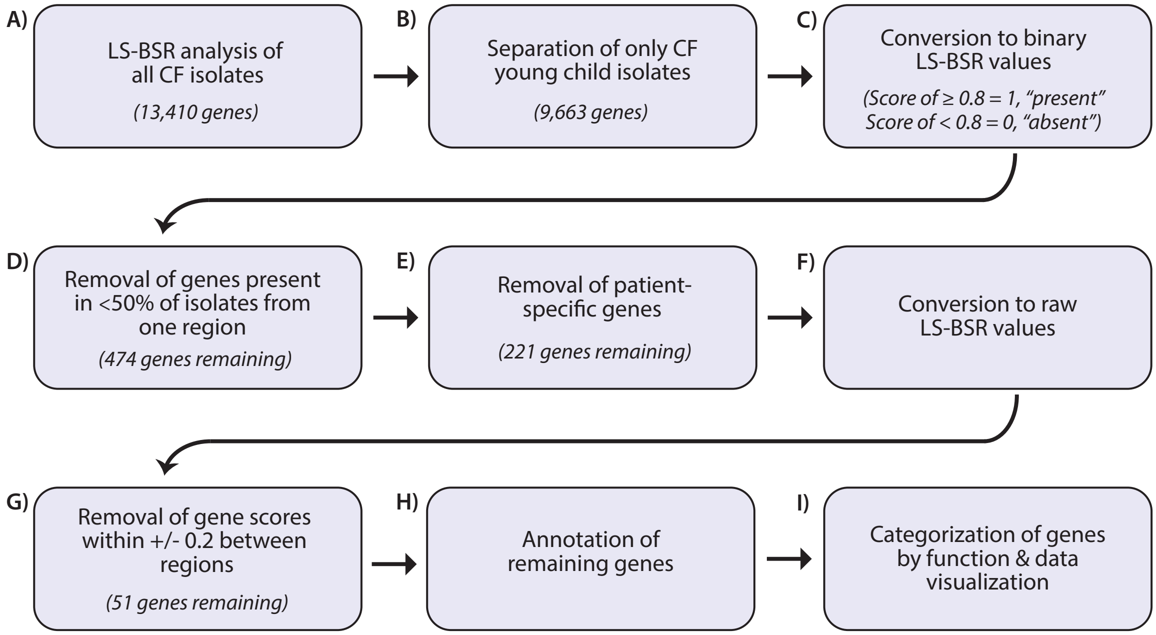

Supplemental Figure 1. Flowchart of the geographical analysis process. Overview of the analysis process for identifying genes influenced by region. Each step is discussed in detail in the results section.

Region-specific Gene Annotations			
Reg.	Gene Call	Annotation	Class
1	CEC100_42_7	O-antigen polysaccharide polymerase Wzy family protein	Polysaccharide
1	CEC100_6_138	hypothetical protein	Hypothetical
1	CEC120_25_37	O-antigen polysaccharide polymerase Wzy family protein	Polysaccharide
1	CEC115_31_26	O-antigen polysaccharide polymerase Wzy family protein	Polysaccharide
1	CEC100_42_1	hypothetical protein	Hypothetical
1	CEC100_42_6	polysaccharide biosynthesis family protein	Polysaccharide
1	CEC100_42_5	glycosyl transferases group 1 family protein	Misc
1	CEC100_42_8	glycosyl transferase 2 family protein	Misc
1	CEC100_42_2	UDP-N-acetyl-2-amino-2-deoxy-D-glucuronate oxidase	Polysaccharide
1	CEC100_42_3	UDP-2-acetamido-3-amino-2,3-dideoxy-D-glucuronate N-acetyltransferase	Polysaccharide
1	CEC36_1_150	hypothetical protein	Hypothetical
1	CEC100_6_172	CP23 domain protein	Misc
1	CEC100_6_164	conserved hypothetical protein	Hypothetical
1	CEC100_6_169	antitoxin VapB	Misc
1	CEC100_6_165	phage Gp37/Gp68 family protein	Phage
1	CEC100_6_170	tRNA(fMet)-specific endonuclease VapC	Misc
1	CEC100_19_41	conserved hypothetical protein	Hypothetical
1	CEC100_42_9	NAD dependent epimerase/dehydratase family protein	Redox
1	CEC31_25_30	glycosyl transferases group 1 family protein	Misc
1	CEC100_17_64	transposase family protein	Transposition
1	CEC100_17_65	putative transposase	Transposition
1	CEC116_231_1	transposase IS66 family protein	Transposition
1	CEC100_62_5	bacterial regulatory, tetR family protein	Regulation
1	CEC100_62_2	conserved hypothetical protein	Hypothetical
1	CEC100_62_3	alpha/beta hydrolase family protein	Misc
2	CEC60_127_2	conserved hypothetical protein	Hypothetical
2	CEC60_140_6	conserved hypothetical protein	Hypothetical
2	CEC60_20_13	conserved hypothetical protein	Hypothetical
2	CEC60_30_12	restriction endonuclease family protein	Regulation
2	CEC60_80_5	conserved hypothetical protein	Hypothetical
2	CEC82_321_2	conserved hypothetical protein	Hypothetical
2	CEC60_22_44	hypothetical protein	Hypothetical
2	CEC60_77_11	conserved hypothetical protein	Hypothetical
2	CEC60_22_45	conserved hypothetical protein	Hypothetical
2	CEC60_77_17	conserved hypothetical protein	Hypothetical
2	CEC60_30_13	AAA domain protein	Replication
2	CEC60_77_14	methyltransferase domain protein	Misc
2	CEC60_80_4	conserved hypothetical protein	Hypothetical
2	CEC60_4_81	uvrD/REP helicase N-terminal domain protein	Replication
2	CEC60_104_11	AAA domain protein	Replication
2	CEC60_4_80	AAA ATPase domain protein	Replication
2	CEC60_22_43	putative lipoprotein Rz1, uncharacterized	Phage
2	CEC60_140_4	HAD hydrolase, IA, variant 1 family protein	Redox
2	CEC82_210_3	hypothetical protein	Hypothetical
2	CEC60_127_3	regulatory protein RepA	Replication
2	CEC60_20_11	fic/DOC family protein	Regulation
3	CEC122_17_29	glyoxalase/Bleomycin resistance /Dioxygenase superfamily protein	Redox
3	CEC122_17_25	FAD binding domain protein	Redox
3	CEC122_17_26	bacterial regulatory, tetR family protein	Regulation
3	CEC122_17_28	bacterial regulatory, tetR family protein	Regulation
3	CEC122_17_30	fumarylacetoacetate (FAA) hydrolase family protein	Metabolism

Supplemental Table 2. Annotation of region-specific genes. Each gene call was annotated automatically, and based on this identification, gene classes were manually assigned. Misc = miscellaneous and is used to describe proteins with functions that could not be clearly defined after a literature search.

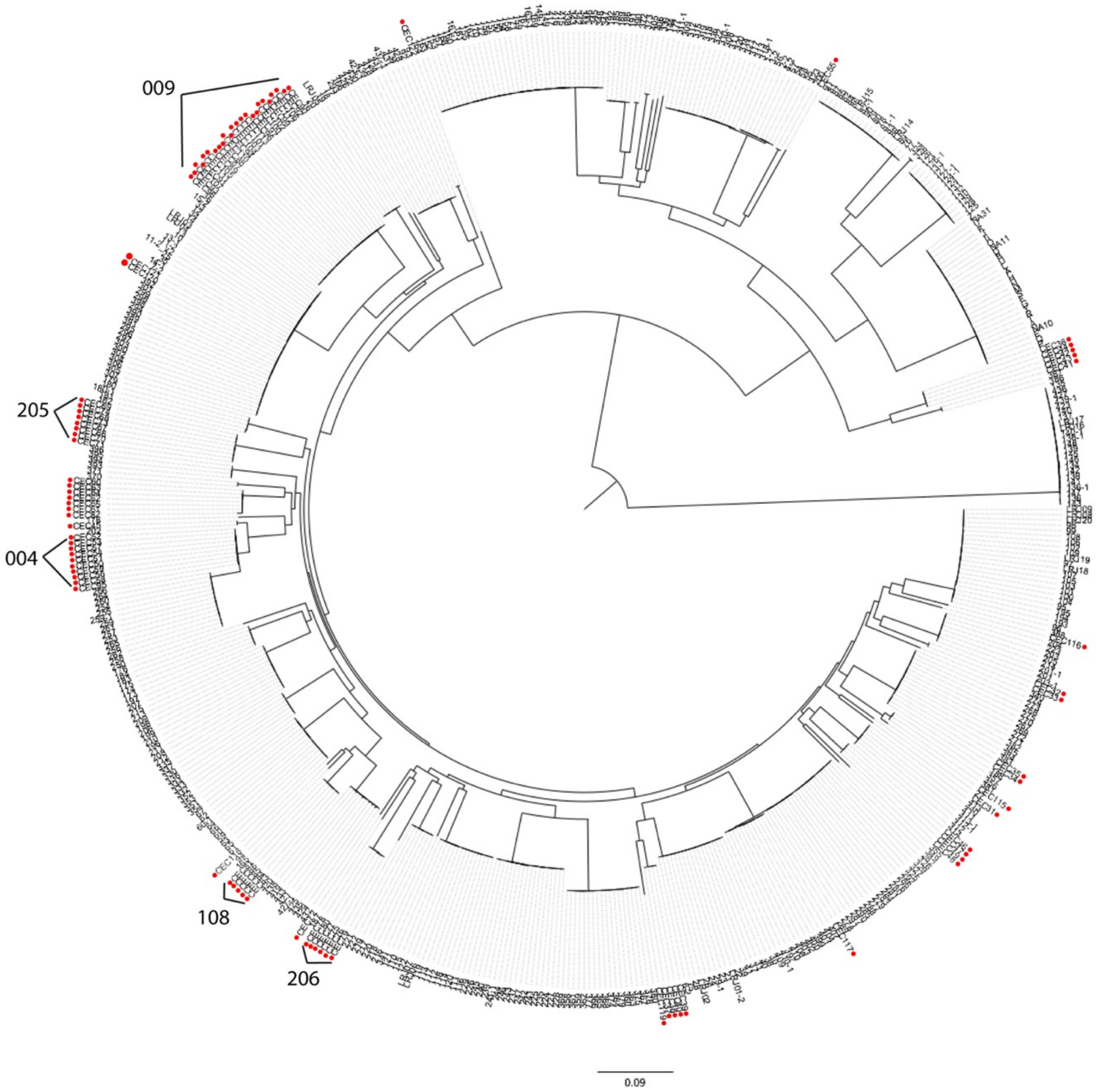

Supplemental Figure 2. Phylogenetic analysis of CF isolates from Denmark and the US. Inferred phylogeny of 90 CF isolates from young children and adults in the U.S. (from this study) and 474 previously-sequenced isolates longitudinally collected from CF patients in Denmark.[255] Isolates from the U.S. are indicated with a red dot. Isolates from a single patient that clustered together are bracketed next to the patient number.

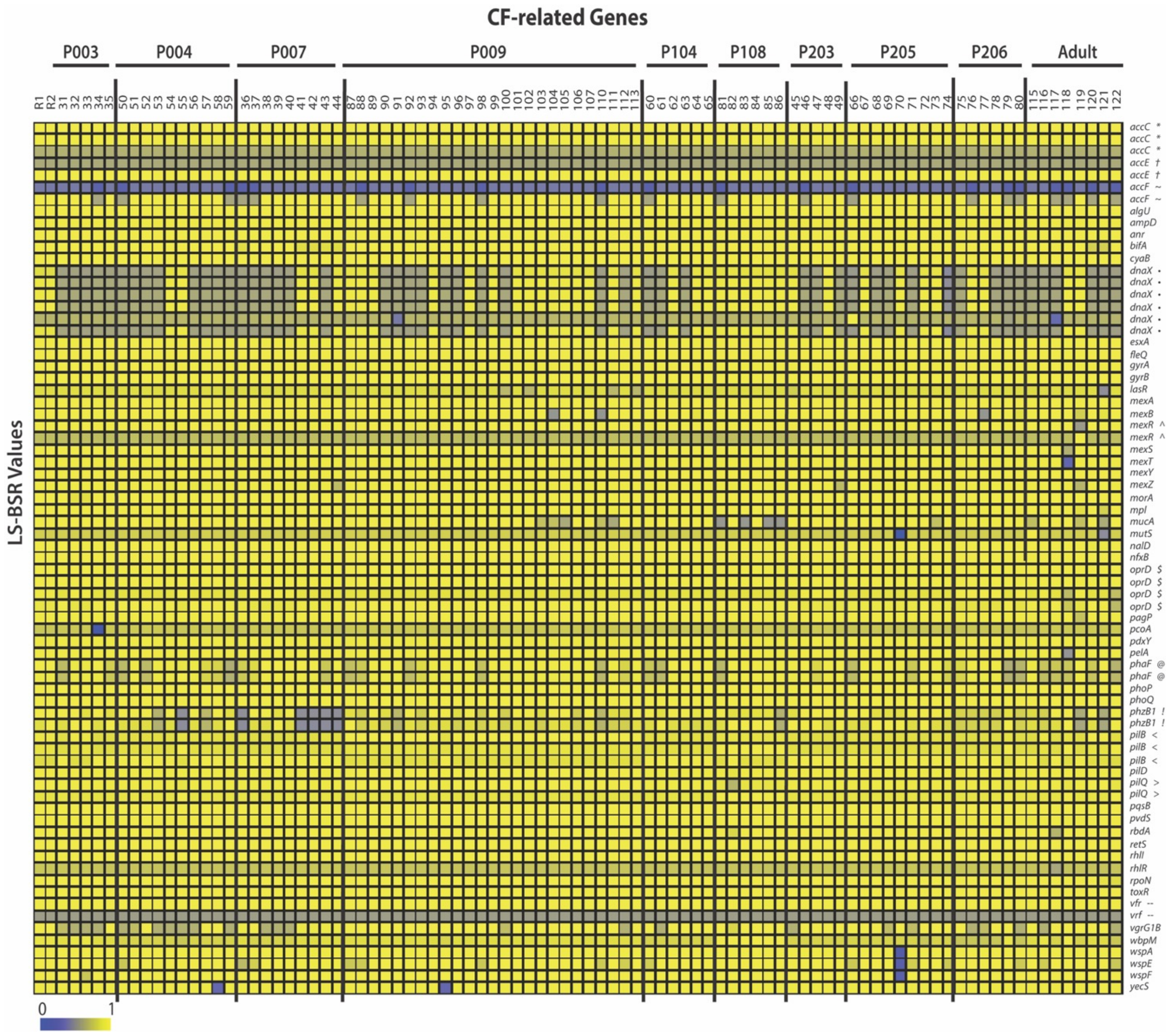

Supplemental Figure 3. LS-BSR analysis of CF-related genes in the CF isolates. CF-related genes were identified based on a literature search. Genes with allelic variants are indicated with symbols.

A.

```
PA0996_pqsA   ACGCTCGGTTTCTGCCGGGCGTTCGCTACGG —//— GGTGGTGCCGACCCAGGTCGAGCAGGCGATCTGCCGT  1260
37            ------------------------------- —//— -------------------------------------  0
38            ------------------------------- —//— -------------------------------------  0
39            ------------------------------- —//— -------------------------------------  0
40            ------------------------------- —//— -------------------------------------  0

PA0996_pqsA   CATCTGCCGGAAGTGAGCGAGGCGGTTCTGGTTCCTACCTGCCGGCTGCACGACGGCTTG  1320
37            -------------------------------------------------ACGACGGCTTG  11
38            -------------------------------------------------ACGACGGCTTG  11
39            -------------------------------------------------ACGACGGCTTG  11
40            -------------------------------------------------ACGACGGCTTG  11
                                                               ***********

PA0996_pqsA   CGTCCGACCCTGTTCGTCACCCTGGCCACTCCGCTGGACGACAACCAGATCCTGCTGGCG  1380
37            CGTCCGACCCTGTTCGTCACCCTGGCCACTCCGCTGGACGACAACCAGATCCTGCTGGCG  71
38            CGTCCGACCCTGTTCGTCACCCTGGCCACTCCGCTGGACGACAACCAGATCCTGCTGGCG  71
39            CGTCCGACCCTGTTCGTCACCCTGGCCACTCCGCTGGACGACAACCAGATCCTGCTGGCG  71
40            CGTCCGACCCTGTTCGTCACCCTGGCCACTCCGCTGGACGACAACCAGATCCTGCTGGCG  71
              ************************************************************

PA0996_pqsA   CAGCGCATCGACCAGCATCTCGCCGAACAGATTCCCTCGCACATGCTGCCCAGCCAATTG  1440
37            CAGCGCATCGACCAGCATCTCGCCGAACAGATTCCCTCGCACATGCTGCCCAGCCAATTG  131
38            CAGCGCATCGACCAGCATCTCGCCGAACAGATTCCCTCGCACATGCTGCCCAGCCAATTG  131
39            CAGCGCATCGACCAGCATCTCGCCGAACAGATTCCCTCGCACATGCTGCCCAGCCAATTG  131
40            CAGCGCATCGACCAGCATCTCGCCGAACAGATTCCCTCGCACATGCTGCCCAGCCAATTG  131
              ************************************************************

PA0996_pqsA   CATGTGCTGCCGGCCTTGCCGCGCAACGACAACGGCAAGTTGGCGCGCGCCGAGCTGCGC  1500
37            CATGTGCTGCCGGCCTTGCCGCGCAACGACAACGGCAAGTTGGCGCGCGCCGAGCTGCGC  191
38            CATGTGCTGCCGGCCTTGCCGCGCAACGACAACGGCAAGTTGGCGCGCGCCGAGCTGCGC  191
39            CATGTGCTGCCGGCCTTGCCGCGCAACGACAACGGCAAGTTGGCGCGCGCCGAGCTGCGC  191
40            CATGTGCTGCCGGCCTTGCCGCGCAACGACAACGGCAAGTTGGCGCGCGCCGAGCTGCGC  191
              ************************************************************

PA0996_pqsA   CACCTGGCCGACACCCTTTATCACGACAACCTTCCGGAGGAACGGGCATGTTGA  1554
37            CACCTGGCCGACACCCTTTATCACGACAACCTTCCGGAGGAACGGGCATGTTGA  245
38            CACCTGGCCGACACCCTTTATCACGACAACCTTCCGGAGGAACGGGCATGTTGA  245
39            CACCTGGCCGACACCCTTTATCACGACAACCTTCCGGAGGAACGGGCATGTTGA  245
40            CACCTGGCCGACACCCTTTATCACGACAACCTTCCGGAGGAACGGGCATGTTGA  245
              ******************************************************
```

B.

```
psD      MGNPILAGLGFSLPKRQVSNHDLVGRINTSDEFIVERTGVRTRYHVEPEQAVSALMVPAA   60
36       MGNPILAGLGFSLPKRQVSNHDLVGRINTSDEFIVERTGVRTRYHVEPEQAVSALMVPAA   60
         ************************************************************

psD      RQAIEAAGLLPEDIDLLLVNTLSPDHHDPSQACLIQPLLGLRHIPVLDIRAQCSGLLYGL   120
36       RQAIEAAGLLPEDIDLLLVNTLSPDHHDPSQACLIQPLLGLRHIPVLDIRAQCSGLLYGL   120
         ************************************************************

psD      QMARGQILAGLARHVLVVCGEVLSKRMDCSDRGRNLSILLGDGAGAVVVSAGESLEDGLL   180
36       QMARGQILAGLARHVLVVCGEVLSKRMDCSDRGRNLSILLGDGAGAVVVSAGESLEDGLL   180
         ************************************************************

psD      DLRLGADGNYFDLLMTAAPGSASPTFLDENVLREGGGEFLMRGRPMFEHASQTLVRIAGE   240
36       DLRLGADGNYFDLLMTAAPGSASPTFLDENVLREGGGEFLMRGRPMFEHASQTLVRIAGE   240
         ************************************************************

psD      MLAAHELTLDDIDHVICHQPNLRILDAVQEQLGIPQHKFAVTVDRLGNMASASTPVTLAM   300
36       MLVAHELTLDDIDHVICHQPNLRILDAVQEQLGIPQHKFAVTVDRLGNMASASTPVTLAM   300
         **.*********************************************************

psD      FWPDIQPGQRVLVLTYGSGATWGAALYRKPEEVNRPC   337
36       FWPDIQPGQRVLVLTYGSGATWGAALYRKPEEVNRPC   337
         *************************************
```

Supplemental Figure 4. (A) pqsA alignment. Multiple alignment of pqsA sequences from isolates #37-40, all from patient 007, compared to reference isolate PAO1 reveals a significant gene truncation (loss of the first ~1,300 nucleotides). **(B)** pqsD alignment. Alignment of translated protein sequences of pqsD from isolate #36 from patient 007 compared to reference isolates PAO1 revealed no amino acid sequence dissimilarity. * indicates residue conservation.

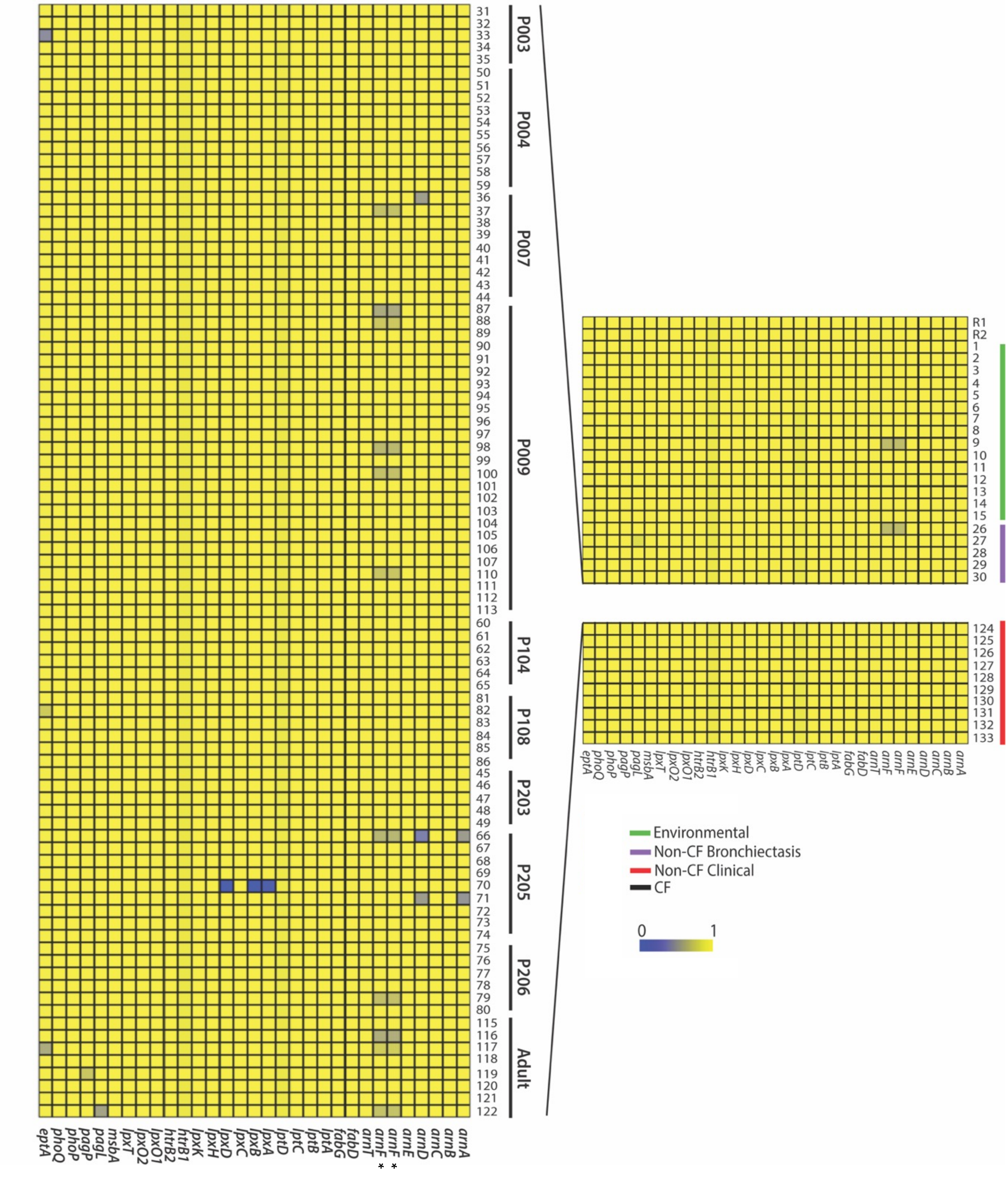

Supplemental Figure 5. LS-BSR analysis of lipid A synthesis and modification genes. LS-BSR scores of lipid A synthesis and modification genes were compared between all isolates. LS-BSR score of 1 = 100% sequence identity (yellow), 0 = 0% sequence identity (blue). Allelic variation in *arnF* is noted with *.

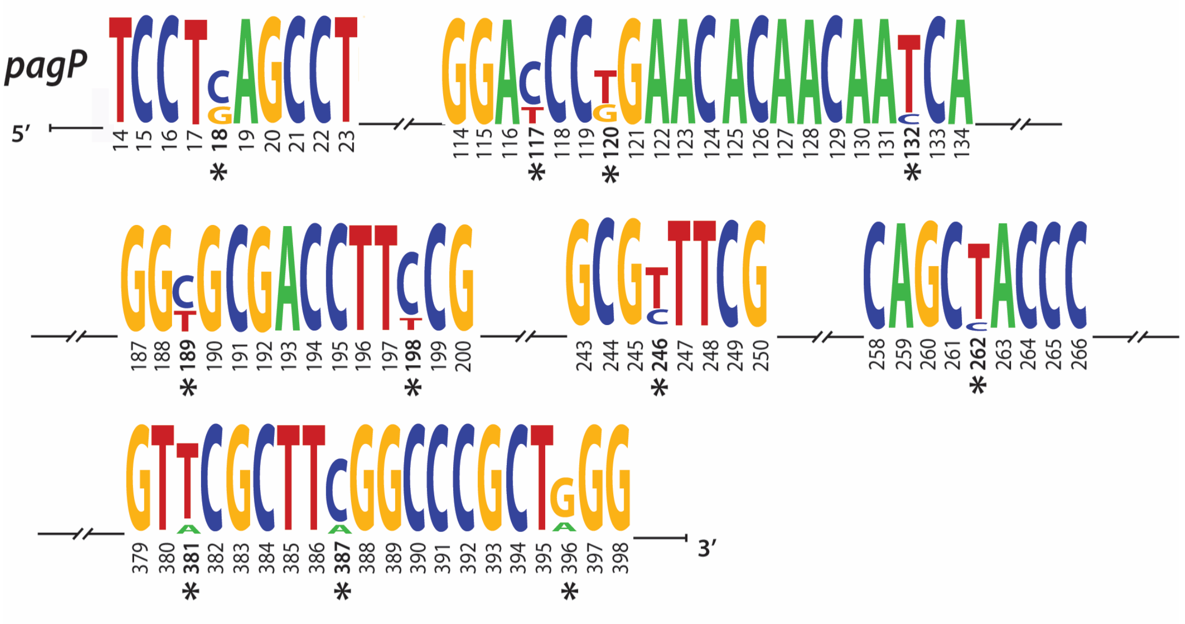

Supplemental Figure 6. All *pagP* sequences from CF isolates were aligned using ClustalW and sequence logos were generated using WebLogo (details in Materials and Methods). * indicates sites of dissimilarity.

	P003					P004										P007								
m/z	31	32	33	34	35	50	51	52	53	54	55	56	57	58	59	36	37	38	39	40	41	42	43	44
1366	+	+				+	+	+	+	+	+	+		+	+	+	+	+	+	+	+	++	+	+
1417	+	++				+	+	+	+	+		+		+	+	+	+	+	+	+	+			
1446	++++	++++	++++	++++	++++	++++	++++	++++	++++	++++	++++	++++	++++	++++	++++	++++	++++	++++	++++	++++	++++	++++	++++	++++
1577		+																						
1616	++	++				++	++	++	++	++	++	++	++	++	++	+	+	++	++	++	++	+		+
1684		++	+	+	+	+	+	+	+	+		+		+	+	+		+	+					

	P009																								
m/z	87	88	89	90	91	92	93	94	95	96	97	98	99	100	101	102	103	104	105	106	107	110	111	112	113
1366	++	+++	++	+++	+	+	+	+	++	++++	++	++	+++	++	+	+++	++++	++++	++++	++	++++	++	++	++	++
1417																		+		+		+			
1446	++++	++++	++++	++++	++++	++++	++++	++++	++++	+++	++++	++++	++++	++++	++++	++++	++++	++	++	++++	++++	++++	++++	++++	++++
1577																		+							
1616	++	++	++	++	+	+	+	+	++	++	++	++	+++	+++	+++	++++	++++	++	++	+++	++	+++	++	+++	+++
1684			+																						

	P104						P108						P203					P205						
m/z	60	61	62	63	64	65	81	82	83	84	85	86	45	46	47	48	49	66	67	68	69	70	71
1366	+	+	+	++	++	++	++	++	++++	+++	++	+++	+	+	+	+	+	+	+++	+	+	+	+
1417							+	+	+		+								+				
1446	++++	++++	++++	++++	++++	++++	++++	++++	++++	++++	++++	++++	+++	++++	++++	++++	++++	++++	++++	++++	++++	++++	++++
1577											+								+				
1616	++	++	++	++	++	++	+++	+++	++	++	+++	+++	+	++	++	++	++	+	++	++	+	++	+
1684								+					+							+	+	+	

	P205			P206						CF Adult						
m/z	72	73	74	75	76	77	78	79	80	115	116	117	118	120	121	122
1366	+	+	+	+	++	+	+	+	+	++	++	++++	+	++	++	++
1417	+									+					+	+
1446	++++	++++	++++	++++	++++	++++	++++	++++	++++	+	++++	+++	++	+++	++++	++++
1577																
1616	++	++	+	++	++	++	++	++	+++	++++	+	+	++++	++	++	++
1684	+			+				+			+		+			

++++	100% relative intensity
+++	75% relative intensity
++	50% relative intensity
+	≤ 25% relative intensity

Supplemental Figure 7. Lipid A structures were analyzed from MALDI spectra in negative ion mode. The highest peak was set at 100% relative intensity and the relative abundance of all other peaks were measured in relation to the maximum peak. *See Supplemental Table 4 for m/z values and their correlated lipid A structures.*

Hypothetical m/z	Predicted Structure	Expected Enzymes Involved in Modification
**1350**	Mono-phosphorylated, penta-acylated, no 2-hydroxylation	PagL
**1366**	Mono-phosphorylated, penta-acylated, one 2-hydroxylation	LpxO1/2†, PagL
**1497**	Mono-phosphorylated, penta-acylated, one 2-hydroxylation, Ara4N addition	*arn* operon, LpxO1/2†, PagL
**1430**	Bis-phosphorylated, penta-acylated, no 2-hydroxylation	PagL
**1446**	Bis-phosphorylated, penta-acylated, one 2-hydroxylation	PagL, LpxO1/2†
**1462**	Bis-phosphorylated, penta-acylated, two 2-hydroxylations	PagL, LpxO1, LpxO2
**1577**	Bis-phosphorylated, penta-acylated, one 2-hydroxylation, Ara4N addition	PagL, LpxO1/2†, arn genes
**1600**	Bis-phosphorylated, hexa-acylated, no 2-hydroxylation	
**1616**	Bis-phosphorylated, hexa-acylated, one 2-hydroxylation	LpxO1/2†
**1632**	Bis-phosphorylated, hexa-acylated, two 2-hydroxylations	LpxO1, LpxO2
**1604**	Mono-phosphorylated, hexa-acylated, one 2-hydroxylation	LpxO1/2†, PagP
**1668**	Bis-phosphorylated, hexa-acylated, no 2-hydroxylation	PagL, PagP
**1684**	Bis-phosphorylated, hexa-acylated, one 2-hydroxylation	PagL, PagP, LpxO1/2†
**1700**	Bis-phosphorylated, hexa-acylated, two 2-hydroxylations	PagL, PagP, LpxO1, LpxO2
**1838**	Bis-phosphorylated, hepta-acylated, no 2-hydroxylation	PagP
**1854**	Bis-phosphorylated, hepta-acylated, one 2-hydroxylation	PagP, LpxO1/2†
**1870**	Bis-phosphorylated, hepta-acylated, two 2-hydroxylations	PagP, LpxO1, LpxO2

Supplemental Table 3. m/z values and their correlated predicted lipid A structures. Predicted lipid A structures are listed with their associated m/z value. Biosynthetic genes involved the observed structure are listed on the right. The observed mono-phosphorylated lipid A may be a result of the extraction procedure, as the phosphate bonds are the most labile. †LpxO1 and LpxO2 are two enzymes that add hydroxyl groups onto lipid A in a site-specific manner; therefore, when loss of hydroxylation is observed by mass spectrometry (-*m/z*16), one cannot determine whether this difference is driven by loss of LpxO1 or LpxO2.

Supplemental Table 4. *P. aeruginosa* gene loci examined in this study. *P. aeruginosa* gene name, reference genome and locus tag are listed.

Supplemental Table 5. LS-BSR data presented in this study. LS-BSR values for all heatmaps in this study are listed.
